# A 22-year experience of surgical management of anorectal melanoma: risk factors for recurrence and death

**DOI:** 10.1007/s00384-025-04861-6

**Published:** 2025-04-22

**Authors:** Richard Sassun, David W. Larson, Annaclara Sileo, Jyi Cheng Ng, Davide Ferrari, Nicholas P. McKenna, William R. G. Perry

**Affiliations:** Division of Colon and Rectal Surgery, Mayo Clinic, 200 First St. Southwest, Rochester, MN 55905 USA

**Keywords:** Anorectal melanoma, Radiotherapy, Immunotherapy, Survival, Recurrence, Margins

## Abstract

**Purpose:**

Anorectal melanoma (ARM) poses a significant challenge due to the lack of established guidelines and a 5-year overall survival rate of less than 20%. The only recognized death risk factors are positive lymph nodes and positive surgical margins. This study aimed to identify the risk factors for local/distant recurrences and death in a 22-year multi-institutional experience.

**Methods:**

All patients who underwent curative surgical resection or were referred to after resection at the Mayo Clinic for non-metastatic ARM (2002–2024) were included. Risk factors for local/distant recurrences, and deaths were assessed through multivariable Cox regression.

**Results:**

Eighty-eight patients were included in the study. Seventy-eight percent of patients had anal melanoma and 22% rectal melanoma. Nineteen percent had clinically positive lymph nodes. The surgical margins were positive in 62% of local surgeries, while they were positive in 13% of radical surgery cases. The first recurrence was often a local recurrence (67%), followed by distant metastasis (21%), with an overall comparable overall survival between the two. Radiotherapy administration, radical surgery, and negative margins were associated with less local recurrence. Clinically positive lymph nodes and local recurrences increased the risk of developing distant metastasis over time. Clinically negative lymph nodes, radiotherapy administration, radical surgery, and negative margins all contributed to a reduced death risk.

**Conclusion:**

Local recurrences in ARM may influence distant metastasis and death more than what was previously believed. Positive surgical margins in local surgery were remarkably high, reaching 62%. Protective factors for local recurrence and death included radical surgery, negative surgical margins, and radiotherapy.

## Introduction

Anorectal melanoma (ARM), comprising less than 1% of all melanomas and 0.1% of anorectal malignancies [1–4], represents a challenge due to its increasing incidence and a five-year overall survival (OS) rate below 20% [5–8]. This low survival rate could be attributed to the aggressive nature of ARM, delay in diagnosis, rapid disease progression, and high metastasis rates compared to other melanoma subtypes [9].

Because of the rarity of the disease and the controversial data in the literature, a consensus on the management of ARM is lacking. Currently, the only curative treatment option for non-metastatic patients is surgery, as radiotherapy and systemic treatments such as chemotherapy and immunotherapy showed limited effect when used alone [10, 11]. Radical operations such as abdominoperineal resection or low anterior resection were historically considered the standard treatments for ARM. However, their role became questioned due to the emergence of local surgery, which showed a comparable OS when negative margins are achieved, simultaneously preserving a good quality of life [4, 6, 12–14].

Achieving negative margins can be challenging in local surgery due to the proximity of the sphincter complex, which should not be damaged to preserve continence. In some cases, ARM may present as a bleeding polypoidal lesion, which can be often mistaken for a hemorrhoid and thus excised in a non-oncological manner. Therefore, the positive margins rate in local surgery is high, ranging from 30 to 73%, which conflicts with the only current recommendation of negative margins and potentially further reducing the OS [10, 14–18]. Radical operations are now seen as excessive for this disease, as many surgeons’ first approach is to be as conservative as possible, avoiding stoma creation and organ resection if deemed feasible. However, every approach should be reconsidered when new therapies, such as immunotherapy, are available.

Given the paucity of extensive institutional data on this rare disease, this study aims to describe the 20-year experience in the treatment of ARM at a quaternary center and to identify the risk factors for recurrences and death.

## Methods

This study was conducted per the “strengthening the reporting of observational studies in epidemiology” (STROBE) guidelines [19]. After Institutional Review Board approval, this study included all patients between January 2002 and April 2024 who underwent surgical resection with curative intent or were surgically or medically treated afterward at the Mayo Clinic enterprise (Minnesota, Florida, Arizona) for non-metastatic ARM. Exclusion criteria were no follow-up and patients who did not undergo any surgery. Patients referred for ARM recurrence were excluded.

Patient demographic data such as age, sex, race, body mass index (BMI), date of diagnosis, symptoms, preoperative tumor characteristics and localization, type of radiotherapy, chemotherapy, or immunotherapy treatment were collected. Peri/post-operative data were reviewed to identify key markers of surgical performance and outcome, including the type of operation performed, need for a second surgery, pathology report, length of hospital stay, number of lymph nodes harvested, number of positive lymph nodes, margins resection status at first and second surgery, tumor size, cancer status, date of recurrences, vital status, and last follow-up date.

Reoperation was defined as patients who underwent a second curative surgical resection. Local surgery (LS) is the term for local excision, while radical surgery (RS) includes abdominoperineal resection (APR) and low anterior resection (LAR). “Definitive surgery” was defined as the type of most recent surgery. The “definitive surgical margin status” refers to the margin status of the definitive surgery. Definitive surgical margin status and definitive surgery were used in the Cox regression analyses.

A comprehensive “systemic therapy” variable was created to limit the number of variables to fit in the multivariate analysis. Immunotherapy regimens included pembrolizumab, ipilimumab, nivolumab, imatinib, pembrolizumab + ipilimumab, ipilimumab + nivolumab, resiquimod, interferon-alpha, or sargramostim. Chemotherapy regimens included temozolomide, temozolomide + cisplatin, or paclitaxel + carboplatin.

Local recurrences were defined as biopsy-proven relapses in the rectum, pelvis, pelvic lymph nodes, iliac lymph nodes, and inguinal lymph nodes (for anal cancers). Distant metastases were similarly defined as biopsy-proven relapses in distant organs.

Categorical variables were reported as frequencies (percent), while continuous variables were reported as mean ± standard deviation (SD) or median [interquartile range] according to their distribution. Missing values were excluded from the descriptive analyses. The chi-square and Fisher tests for categorical variables and Mann–Whitney *U* test for continuous variables were used to compare groups. Cox regression and Kaplan–Meier analyses were conducted to identify risk factors for death and recurrences. All tests were two-sided; a significant difference was considered with an alpha level < 0.05. The statistical analysis utilized BlueSky Statistics software v. 10.3.4 (BlueSky Statistics LLC, Chicago, IL, USA).

## Results

### Demographic and tumor characteristics

**Fig. 1:**
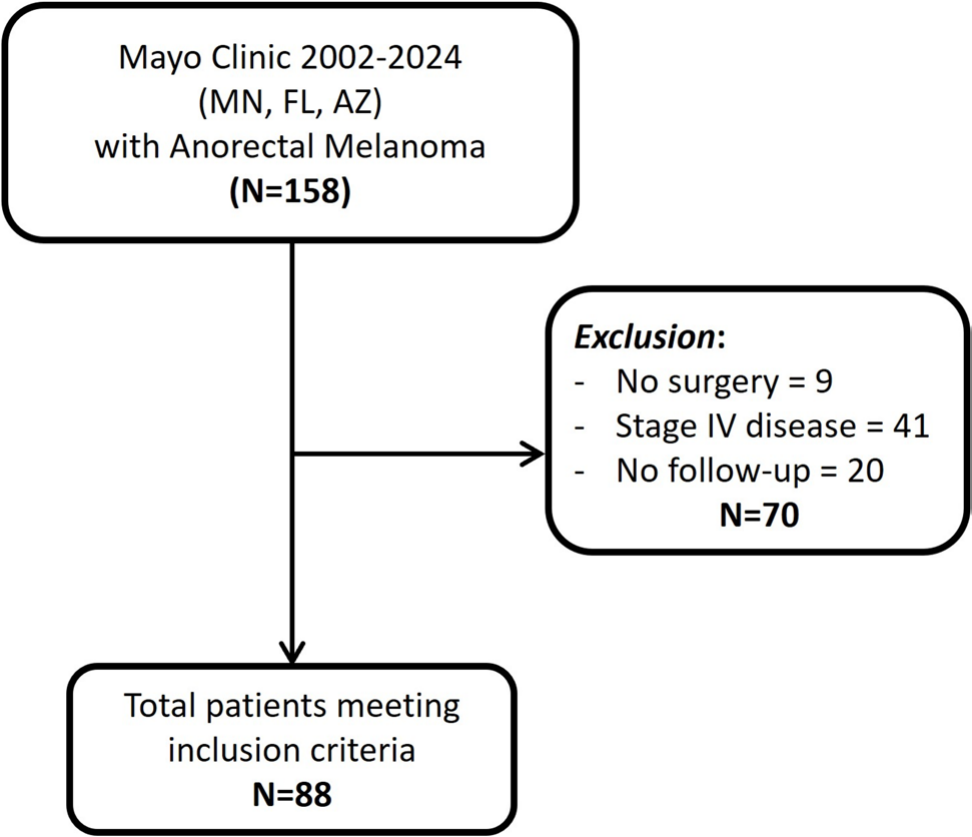
Flowchart of cohort development

After the exclusion criteria, 88 patients were included in the study. The cohort development is depicted in Fig. 1, while demographics and tumor characteristics are summarized in Table 1. Fifty-five (63%) patients were female. The mean age of the cohort was 64 ± 10 years, while the mean BMI was 29.6 (± 6.3) kg/m^2^. The most frequent symptom was rectal bleeding, seen in 70 (80%) patients, followed by hemorrhoid-like protrusions in 7 (8) patients. None of the patients were referred to the Mayo Clinic enterprise due to recurrence.

**Table 1:** Cohort demographic and tumor characteristics

Characteristics	Patients (*n* = 88)
Men, *n* (%)	33 (37)
Age, years, mean (SD)	64 (10)
BMI, kg/m^2^, mean (SD)	29.6 (6.3)
Symptoms, *n* (%)	
Rectal bleeding	70 (80)
Hemorrhoid like	7 (8)
Mass	4 (4)
Incidental finding	7 (8)
Location, *n* (%)	
Rectum	19 (22)
Anal canal	69 (78)
Histology, *n* (%)	
Nodular	3 (3)
Spindle cell	8 (9)
Superficial spreading	2 (2)
Not otherwise specified	75 (85)
Depth of invasion, mm, mean (SD)*	9.7 (4.2)
Number of mitosis/mm2, median (IQR)*	10 (7–17)
Tumor diameter, cm, mean (SD)*	2.4 (0.9)
Multifocal, *n* (%)	17 (81)
Ulcerated, *n* (%)	54 (61)
Lymphovascular invasion, *n* (%)	28 (32)
Positive clinical lymph nodes* n* (%)	17 (19)
Immunochemistry, *n* (%)*	
S-100	57 (92)
MART-1	23 (100)
MELAN-A	46 (52)
VIMENTIN-A	4 (67)
HMB45	42 (96)

^*^Variable contains missing data

The anal canal was the prevalent location of the disease (*n* = 69, 78%), with the remaining cases being in the rectum. Clinically positive lymph nodes were noted in 17 (19%) patients.

### Treatment characteristics and outcomes

**Fig. 2:**
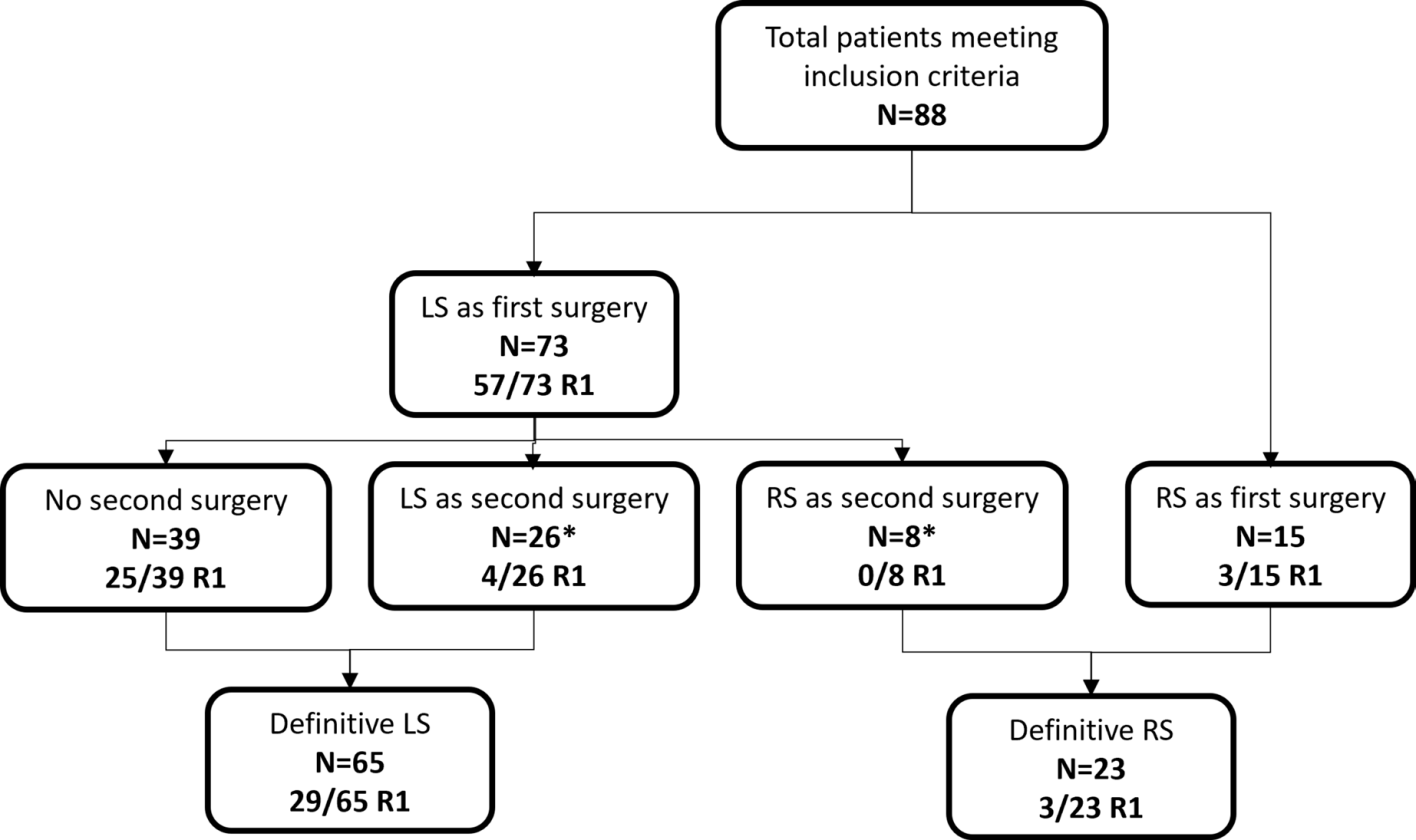
Flowchart of patients’ treatment and surgical margins status

The treatment details are shown in Table 2 and Fig. 2. Of note, 34 (39%) patients required a reoperation. Sixty-five (74%) patients underwent local surgery as the definitive surgery, while 23 (26%) underwent radical surgery (25 APR, one LAR). The definitive surgical margins were positive in 32 (36%) patients: out of the 99 local surgeries (73 first surgeries + 26 reoperations), only 38 surgical margins were negative, indicating 61 patients (62%) with positive surgical margins in local surgery overall. Conversely, out of the 23 radical surgeries, 20 surgical margins were negative, indicating only 3 patients (13%) of positive surgical margins in radical surgery. The median time from first surgery to definitive surgery was 28.5 (20.8–45.0) days. Lymphovascular invasion was present in 28 (32%) patients. Systemic therapy was administrated to 40 (46%) patients (neoadjuvant setting *n* = 3, 3%; adjuvant setting *n* = 37, 42%), while radiotherapy was performed on 20 (23%) patients (neoadjuvant setting *n* = 5, 6%; adjuvant setting n = 15, 17%).

**Table 2:** Treatment details and outcomes

Characteristics	Patients (*n* = 88)
Definitive surgery, *n* (%)	
Local surgery	65 (74)
Radical surgery	23 (26)
Time from 1st surgery to definitive surgery, days, median, (IQR)	28.5 (20.8–45.0)
Definitive surgical margins, *n* (%)	
Negative	56 (64)
Positive	32 (36)
Radiotherapy, *n* (%)	20 (23)
Systemic therapy, *n* (%)	40 (46)
First recurrence	
Local	59 (67)
Distant	18 (21)
None	11 (13)
Follow-up, months, median (IQR)	97.9 (41.9–164.5)
5-year overall survival, % (95% CI)	28 (19–40)

**Fig. 3:**
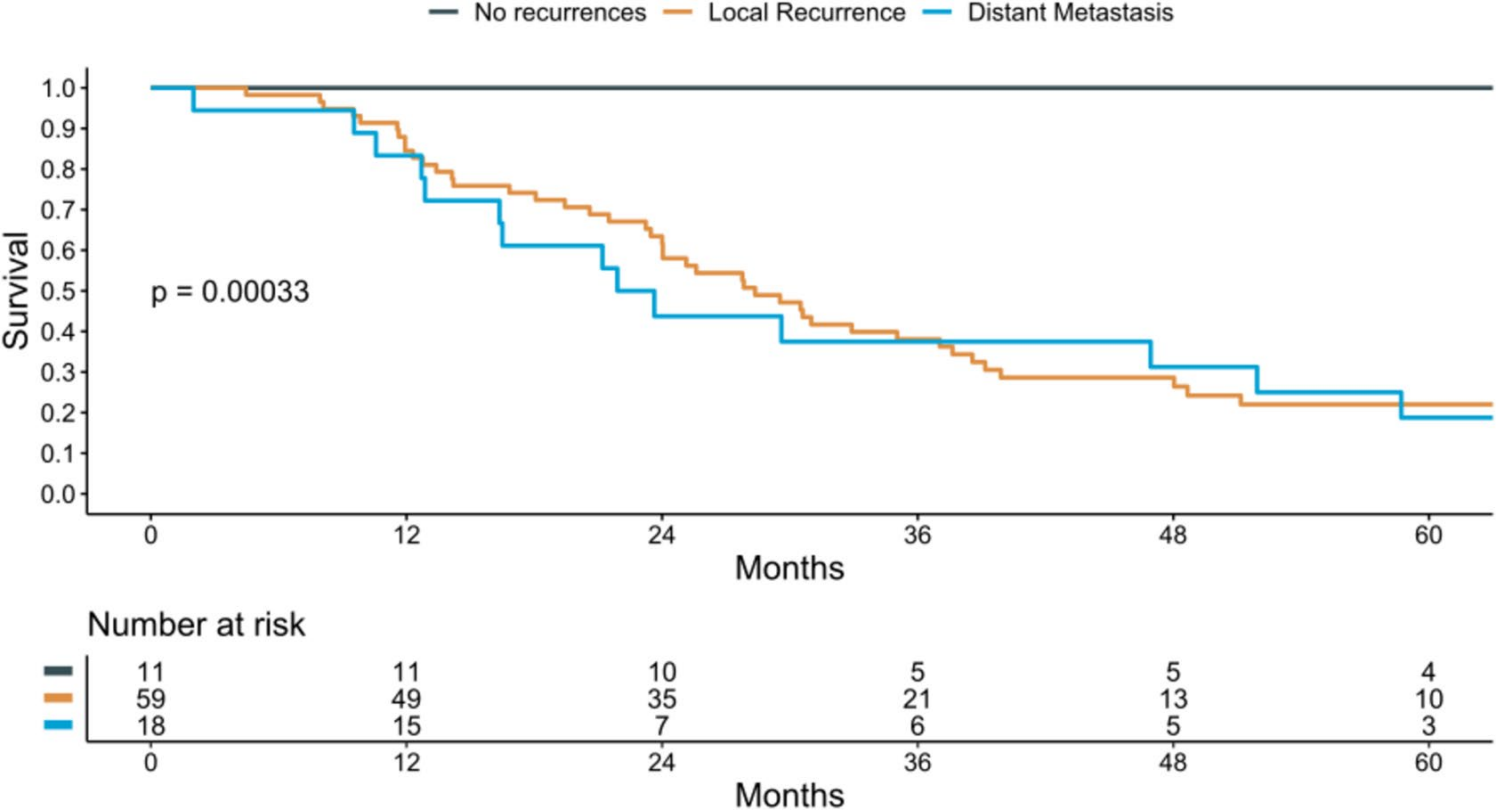
5-year survival Kaplan–Meier analysis based on the pattern of the first recurrence

After a median follow-up of 97.9 (41.9–164.5) months, the first recurrence that the patients presented mainly was a local recurrence (*n* = 59, 67%). Of these, 49 patients subsequently developed distant metastasis, seven patients were lost to follow-up, one patient died of sepsis, and two patients survived after immunotherapy. The median time to local recurrence was 6.1 (2.7–15.6) months, while the median time to distant recurrence for patients with a local recurrence was 14.0 (7.9–26.8) months. Of the 18 (21%) patients that developed distant metastasis as the first recurrence, only one survived five years, while another one was alive in the second year of follow-up. A Kaplan–Meier analysis for 5-year death was performed, revealing comparable death rates between patients with local recurrences or distant metastasis as the first recurrence (Fig. 3). The overall survival rate was 28% (95% confidence interval 19–40%).

### Multivariable analyses

Two multivariable Cox regression analyses were performed to identify risk factors for local and distant recurrences (Table 3). Radiotherapy administration, radical surgery, and negative margins were associated with lower probabilities of local recurrence. A Cox regression model with a time-dependent variable (local recurrence) was used to assess the risk of distant recurrence, accounting also for changes over time. The HR with 95% confidence intervals (CI) revealed that only clinically positive lymph nodes and local recurrences influenced the risk of developing distant metastasis over time.

**Table 3:** Cox regression analyses showing risk factors for local (A) and distant (B) recurrences

	HR (95% confidence interval)	*p*-value
(A) Variable for local recurrences		
Located in the rectum	1.26 (0.62–2.58)	0.515
Ulcerated	0.67 (0.39–1.16)	0.151
Abdominoperineal resection	0.22 (0.09–0.54)	** < 0.001**
Negative Surgical margins	0.36 (0.20–0.63)	** < 0.001**
Radiotherapy performed	0.30 (0.14–0.66)	**0.003**
(B) Variable for distant recurrences		
Located in the rectum	1.16 (0.63–2.13)	0.639
Clinically positive lymph nodes	3.43 (1.76–6.68)	** < 0.001**
Lymphovascular invasion	1.46 (0.84–2.52)	0.178
Abdominoperineal resection	0.91 (0.46–1.80)	0.785
Systemic therapy	1.03 (0.60–1.76)	0.929
Local recurrence	6.85 (3.81–12.33)	** < 0.001**

Similarly, a multivariable Cox regression analysis was performed to identify risk factors for death (Table 4). Clinically negative lymph nodes, radiotherapy administration, radical surgery, and negative margins all contributed to a reduced death probability.

**Table 4:** Cox regression analysis showing risk factors for death

Variable	HR (95% confidence interval)	*p*-value
Located in the rectum	1.75 (0.87–3.52)	0.118
Clinically positive lymph nodes	2.49 (1.12–5.54)	**0.026**
Lymphovascular invasion	1.11 (0.62–1.99)	0.720
Abdominoperineal resection	0.41 (0.18–0.92)	**0.030**
Negative surgical margins	0.58 (0.35–0.99)	**0.044**
Systemic therapy	1.17 (0.68–2.00)	0.581
Radiotherapy performed	0.33 (0.15–0.69)	**0.004**

## Discussion

ARM remains a rare diagnosis for which medical and surgical treatments are not standardized yet, probably contributing to the very low 20% OS at 5 years. This institutional study aimed to describe the 20-year experience in a quaternary center and to identify risk factors for recurrences and death. As a novel paradigm shift, radical surgery and radiotherapy decrease recurrences and mortality. Importantly, these results come from the most extensive institutional cohort currently available in literature.

ARM manifested in 88% of cases with symptoms resembling hemorrhoids, such as rectal bleeding, constipation, and soft tissue protrusion. As a result, careful attention is essential during anal examinations to avoid misdiagnosis. Although local surgery has proven similar OS compared to radical surgery when negative margins are achieved [20–22], local surgery still presents a higher chance of positive margins, leading to worse OS [14, 16, 23]. In fact, local surgery suffers from common deep-margin positive resection, likely because surgeons may stop the dissection at the internal sphincter muscle to avoid damaging it with subsequent incontinence problems [10, 14–16]. Consistent with the literature, positive surgical margins were observed in 62% of the local surgery cases, and only 13% of the radical surgery cases [14, 17, 24].

Notably, most patients experienced some form of recurrence, with local recurrence being the predominant type (67%). Among patients with local recurrence, progression to systematic disease occurred after a median period of 8 months, suggesting that local recurrences may play a more significant role in ARM survival than previously thought and should, therefore, be prevented. This finding challenges the current recommendations, which advocate for local surgery when the surgeon anticipates negative margins. The authors firmly believe that performing local surgery poses a significant risk for local recurrence and subsequent disease progression due to an extreme high positive margin rate and to the nature of the surgery which hinders the harvesting of pelvic lymph node, likely compromised at surgery due to the highly aggressive nature of this tumor. Our hypothesis was supported by the results of the Cox regression analysis, which showed that local surgery, positive definitive margins, and omission of radiotherapy increased the likelihood of local recurrence. Moreover, local recurrence was the strongest predictor of distant metastasis, with a hazard ratio of 6.85. As an expectable consequence, three significant predictors of death in this study were local surgery, positive definitive surgical margins, and clinically positive lymph nodes.

Another important discovery of this study is that radiotherapy could potentially prevent local recurrence and death in ARM. Although a study by Wong et al. [25] revealed no difference in survival rates for patients receiving radiotherapy, it is essential to say that this study was based on a national database, which limits the treatment details and staging for ARM. Another study by Kelly et al. [26] which analyzed only patients with local surgery and adjuvant radiotherapy, showed local disease control of 85%, which is higher than the literature reports of 31–53% [16, 27]. Despite this, their survival rate was still abysmal, possibly due to lymph node micrometastases not being accounted for with local surgery. Concordantly, a growing number of studies are currently supporting the use of radiotherapy in the neoadjuvant or adjuvant treatment of head and neck mucosal melanoma, which shares a prognosis comparable to ARM [28, 29].

One of the hypothetical reasons behind this change of clinical practice, might be that radiotherapy reduces local recurrences which can subsequently cause distant metastases, as suggested by the median period of 8 months between local recurrences and distant metastases.

Even if this study analyzes the current largest ARM cohort from an institutional database, the retrospective nature limits the results. However, conducting clinical trials for such rare diseases is challenging. Although the United Kingdom guidelines were published in 2020 [21], the lack of adherence internationally represents a known major problem for generalization of management and results. Therefore, the tumor’s thickness/pT stage and the mitosis count per mm.^2^ were not assessed for every patient and could not be used for multivariable analysis. Lastly, immunotherapy and chemotherapy were grouped together under a single “systemic therapy” variable, which makes it difficult to assess their individual impact on outcomes. However, studies to date have shown no significant difference in OS between the two [30, 31].

In conclusion, this study highlights a pattern for disease progression in ARM, suggesting that local recurrences may influence distant metastasis and mortality more than what was previously believed. Positive surgical margins in local surgery were remarkably high, reaching 62%. Protective factors for local recurrence and death included negative surgical margins, radical surgery, and radiotherapy, suggesting them as primary objective and treatment for this disease.

## Data Availability

No datasets were generated or analysed during the current study.
